# Maternal perspectives on Multiple Micronutrient Supplementation (MMS) in Indonesia: a cross-sectional study of knowledge, attitudes, and acceptance

**DOI:** 10.1186/s12889-025-24885-5

**Published:** 2025-11-19

**Authors:** Mohammed Alfaqeeh, Auliya A. Suwantika, Maarten J. Postma, Annisa Dewi Nugrahani, Rizka Ayu Setyani, Neily Zakiyah

**Affiliations:** 1 Doctoral Program of Pharmacy, Faculty of Pharmacy, Universitas Padjadjaran, Bandung, Indonesia; 2 Department of Pharmacology and Clinical Pharmacy, Faculty of Pharmacy, Universitas Padjadjaran, Bandung, 45363 Indonesia; 3 Center of Excellence for Pharmaceutical Care Innovation, Universitas Padjadjaran, Bandung, Indonesia; 4 Center for Health Technology Assessment, Universitas Padjadjaran, Bandung, Indonesia; 5 Department of Health Sciences, University Medical Center Groningen, University of Groningen, Groningen, The Netherlands; 6 Department of Economics, Econometrics and Finance, Faculty of Economics and Business, University of Groningen, Groningen, The Netherlands; 7 Doctoral Programme, Faculty of Medicine, Universitas Padjadjaran, Bandung, Indonesia; 8 Department of Obstetrics and Gynecology, Faculty of Medicine, Universitas Padjadjaran, Bandung, Indonesia; 9 Bachelor of Midwifery and Professional Midwifery Education, Faculty of Medicine, Universitas Sebelas Maret, Surakarta, Indonesia

**Keywords:** Multiple micronutrient supplementation, Pregnancy, Maternal health, Antenatal care

## Abstract

**Background:**

Multiple Micronutrient Supplementation (MMS) is recommended globally to improve maternal nutrition and pregnancy outcomes. As Indonesia transitions from iron-folic acid to MMS, understanding the perspectives of pregnant women is critical for effective implementation. However, evidence on pregnant women’s perceptions and acceptance of MMS remains limited. This study aims to assess the levels and determinants of knowledge, attitude, and acceptance (KAA) of MMS among pregnant women in Indonesia.

**Methods:**

A nationwide cross-sectional study was conducted using a validated online survey. Participants were recruited via digital outreach and midwife networks. Descriptive statistics and multivariable logistic regression were used to assess KAA levels and predictors, reported as adjusted odds ratios (aOR) with 95% confidence intervals (CI).

**Results:**

Most participants had a positive attitude (90.1%), 62.7% had good knowledge, and 66.5% reported high acceptance. Poor knowledge was less likely among women aged 25–34 (aOR: 0.27; CI: 0.08–0.89), those with junior high (aOR: 0.46; CI: 0.25–0.85), senior high (aOR: 0.28; CI: 0.16–0.49), or higher education (aOR: 0.32; CI: 0.18–0.57), incomes of 1–3 million (aOR: CI: 0.64; 0.42–0.98), 3–5 million (aOR: 0.56; CI: 0.34–0.87), or > 5 million (aOR: 0.52; CI: 0.33–0.83), and those in the first (aOR: 0.22; CI: 0.14–0.35) or second trimester (aOR: 0.30; CI: 0.20–0.45). Negative attitude was less likely with income of 3–5 million (aOR: 0.46; CI: 0.46–0.89), first (aOR: 0.09; CI: 0.02–0.39) or second trimester (aOR: 0.04; CI: 0.01–0.30), and good knowledge (aOR: 0.52; CI: 0.33–0.82), but more likely among employed women (aOR: 1.60; CI: 1.02–2.49). Low acceptance was more likely in the second trimester (aOR: 2.13; CI: 1.34–3.37), but less likely with good knowledge (aOR: 0.25; CI: 0.18–0.34).

**Conclusion:**

While attitudes toward MMS were largely positive, gaps in knowledge and acceptance persist. Improving knowledge was consistently associated with better attitude and higher acceptance. Strengthening early antenatal education is essential to improving informed acceptance and effective integration of MMS into maternal care in Indonesia.

**Supplementary Information:**

The online version contains supplementary material available at 10.1186/s12889-025-24885-5.

## Introduction

Maternal nutrition is a foundational determinant of maternal and child health [1]. During pregnancy, the demand for essential nutrients increases significantly to support fetal growth, placental development, and maternal physiological changes [2]. Inadequate nutritional intake during this critical period is associated with a range of adverse outcomes, including maternal anemia, preterm birth, intrauterine growth restriction, low birth weight, and long-term developmental deficits in children [3, 4]. Micronutrient deficiencies, often referred to as “hidden hunger”, are pervasive in low- and middle-income countries (LMICs), where access to diverse, nutrient-rich foods may be limited [5, 6]. A recent study found that initiating a multi-micronutrient supplement before conception and continuing it throughout pregnancy led to a 56% decrease in the rate of preterm births [7]. According to a systematic review, an estimated 15 million babies are born prematurely each year [8]. Additionally, around 20.5 million infants are born with low birth weight, with 91% of these cases occurring in LMICs [9]. These infants face a heightened risk of stunted growth, delayed cognitive development, and increased chances of cardiovascular conditions in adulthood [10, 11]. These challenges have prompted increasing global interest in more comprehensive nutritional interventions, such as Multiple Micronutrient Supplementation (MMS), which offers a promising strategy to address the cumulative burden of maternal and neonatal micronutrient deficiencies in LMICs [12].

MMS is an evidence-based intervention designed to address micronutrient deficiencies during pregnancy [13, 14]. MMS was developed by the United Nations International Multiple Micronutrient Antenatal Preparation (UNIMMAP) to meet the nutrient requirements of pregnant women, even in low-resource settings, and support their increased nutritional needs. MMS provides a single tablet that contains a broader spectrum of essential vitamins and minerals. These include 10 vitamins (A, C, D, E, thiamine, riboflavin, niacin, B6, folic acid, and B12) and five minerals (iron, zinc, iodine, copper, and selenium) [15]. Key nutrients, such as zinc, folate, niacin, riboflavin, and vitamins B6 and B12, support early gestation processes like implantation and organogenesis [16]. Zinc and vitamin D are essential for placental function, while iron and iodine are vital for fetal development [17]. A recent modeling study estimated that MMS has the potential to prevent an additional 7 to 28 million infant deaths and disabilities across 32 LMICs [18].

In Indonesia, anemia remains a significant public health issue, with approximately half of all pregnant women affected [19]. Although new studies show a slight decrease in prevalence, anemia still poses a significant concern for maternal health [20, 21]. Since 2014, the Indonesian government has initiated the Iron-Folic Acid Supplements (IFAS) program to prevent and treat anemia among adolescent girls [22]. This program provides supplements containing 60 mg of elemental iron and 400 µg of folic acid, distributed free of charge through community health centers, sub-health centers, and school-based platforms. While IFAS has been moderately effective in reducing anemia, it does not adequately address the broader spectrum of micronutrient deficiencies that commonly affect pregnant women [23]. Recognizing these limitations, the Ministry of Health has initiated a gradual transition toward MMS as of October 2024 [24]. This shift is currently being piloted and scaled up in selected regions, but implementation remains uneven, and pregnant women's awareness, perceptions, and willingness to use MMS are not yet well understood.

Although the clinical benefits of MMS have been well established [25], including through the landmark SUMMIT trial in Lombok, Indonesia [26], few studies have explored pregnant women’s understanding, perceptions, and acceptance of MMS [23, 27], To address this gap, the present study aimed to assess maternal knowledge, attitudes, and acceptance (KAA) of MMS, explore how these vary across key sociodemographic factors, and examine whether knowledge and attitudes independently predict acceptance among pregnant women in Indonesia. To the best of our knowledge, this is the first study in Indonesia to investigate these dimensions collectively from the perspective of pregnant women. The findings are expected to provide critical insights to support the development of culturally sensitive communication approaches and help prioritize targeted strategies to enhance maternal acceptance and uptake of MMS at scale.

## Materials and methods

This study was conducted in accordance with the CROSS (Checklist for Reporting of Survey Studies) guidelines [28]. A detailed protocol was developed before implementation to guide the research process and ensure consistency, although it was not published. The completed CROSS checklist is provided in Multimedia Appendix 1 (Tables S1).

### Study design

A cross-sectional study was conducted using an online survey targeting pregnant women in Indonesia. Women who were currently pregnant, residing in Indonesia, aged 18 years or older, and who had given their consent to participate were eligible for inclusion. Participants who did not complete the questionnaire were excluded from the final analysis. A convenience sampling method was used to recruit participants through digital platforms, including pregnancy-related social media groups, and networks coordinated by local midwives and maternal health advocates. While this approach enabled broad outreach and rapid data collection, it may have introduced selection bias, as the sample may not fully represent all pregnant women in Indonesia.

### Sample size

The required sample size was calculated using Slovin’s formula [29], based on a population of 4.8 million pregnant women in Indonesia [30, 31]. Using a 5% margin of error and anticipating a 10% rate of unusable responses, the minimum required sample size was calculated to be 445 participants.

### Study instrument

The questionnaire was developed based on global and national guidelines related to maternal nutrition, including United Nations (UN) guidelines for assessing nutrition-related tools [32], Indonesian maternal health guidelines [33], and evidence-based psychological theories on health behaviors [34]. It was also informed by a review of relevant literature and existing tools in similar contexts to ensure relevance and evidence-based structure [35–39]. The instrument was specifically developed for this study in the Indonesian language to ensure clarity and cultural relevance for the target population. An English-language version of the questionnaire is provided in Multimedia Appendix 2.

The questionnaire comprised four domains: participant characteristics, knowledge, attitudes, and acceptance of MMS. The participant characteristics domain gathered demographic and background information, including age, educational level, employment status, marital status, monthly household income, residential area, gestational trimester, and gravidity. These items were assessed using closed-ended and short open-ended questions. The knowledge domain was measured through nominal-scale questions with response options such as “Yes,” “No,” and “Don’t know/Not sure,” as well as multiple-choice questions. These captured awareness of MMS, its nutrient content, intended use during pregnancy, recommended timing and frequency of use, and potential side effects. Attitudes toward MMS were assessed using a five-point Likert scale, ranging from “strongly disagree” to “strongly agree,” to evaluate beliefs about the benefits, barriers, and preferences for supplements over natural food sources. The acceptance domain included response options (“Yes,” “No,” “Not sure”) and multiple-response items to assess willingness to consume MMS if provided free or at cost, readiness to replace current supplements with MMS, and likelihood of recommending it to others. 

The draft questionnaire was reviewed by a panel of four experts in maternal health, public health, nutrition, and survey methodology to ensure content validity. Each item was rated for relevance, and feedback was used to refine question phrasing and structure [40, 41]. The individual content validity index (I-CVI) was calculated based on Lynn’s criteria, which recommend an I-CVI of 1.0 for panels of 3–5 experts [42]. Two rounds of I-CVI calculation were conducted. Face validity was assessed through a pretest with 30 pregnant women to evaluate clarity and ease of understanding [43]. Additionally, a pilot study was conducted with 200 participants not included in the final analysis to evaluate the instrument’s reliability and overall validity [44].

### Data collection

Data collection was conducted between March and May 2025. The questionnaire was administered online using the Google Forms platform to facilitate broad accessibility. Participants were recruited through multiple digital channels, including pregnancy-related social media platforms, announcements in healthcare facilities, and community outreach programs targeting pregnant women. The research team collaborated with the Indonesian Society of Obstetricians and Gynecologists (*POGI*) and the Indonesian Society of Maternal–Fetal Medicine (*HIMPISI*), whose networks helped facilitate broader outreach across different regions. Additionally, a small group of trained midwives was engaged to support community-level dissemination of the survey link, particularly in areas with limited online reach. These midwives provided information about the study and referred eligible participants to the online form, but were not involved in data collection or in influencing participants' responses. Participants were required to read a consent statement outlining the study's objective, the voluntary nature of participation, and assurances of anonymity and confidentiality. Informed consent was indicated by checking a box confirming that they had read, understood, and voluntarily agreed to participate in the study.

### Ethical approval

This study was conducted in accordance with the principles of the Declaration of Helsinki and received ethical approval from the Research Ethics Committee of Universitas Padjadjaran (Approval No. 390/UN6.KEP/EC/2025). On the first page of the online survey, participants were provided with an introduction to the study. To proceed, participants were required to read a consent statement outlining the study's objectives, the voluntary nature of participation, and assurances of anonymity and confidentiality. Participants indicated their informed consent by checking a box that confirmed they had read and understood the information and voluntarily agreed to participate in the study.

### Data analysis

In the pilot study, item validity was assessed by calculating the Pearson's correlation coefficient between each item and the total domain score [45]. An item was considered valid if the correlation was statistically significant at *p* < 0.05. Reliability testing was conducted using Cronbach’s alpha, with a threshold of 0.60 or higher indicating an acceptably reliable item [46, 47].

The level of KAA regarding MMS was determined by calculating the total score for each domain. The questionnaire consisted of 23 items in total: 8 demographic questions and 15 items assessing KAA of MMS. The knowledge section included 6 items, each correct answer was awarded 1 point, while incorrect responses or uncertainty were scored as 0, resulting in a maximum possible score of 6 [48]. The attitude domain comprised 5 items, each rated on a five-point Likert scale (1 = strongly disagree to 5 = strongly agree), with a maximum total score of 25 [49]. The acceptance section contained 4 items scored as 1 for affirmative responses and 0 for negative or uncertain ones, with a maximum possible score of 4 [50]. Scores in each domain were converted into percentages for interpretation and classification. For analysis, percentages below 50% were classified as poor/low KAA, while those of 50% or above were classified as good/high KAA [51–54].

Descriptive statistics were used to summarize sociodemographic characteristics. For the knowledge and acceptance domains, the percentage of participants providing correct or affirmative responses for each item was reported. For the attitude domain, response distributions were presented as percentages based on the number of participants selecting each option on the Likert scale. Most of the variables were collapsed to ensure each category had enough data for a more meaningful analysis [55]. Chi-square tests were used to examine univariate associations across all included domains [56]. Variables that showed a significant association with KAA (*p* < 0.25) in the bivariate analysis were included in a multivariable binary logistic regression model [57]. We analyzed factors associated with low acceptance of MMS, as well as risk factors linked to poor maternal knowledge and negative attitudes toward MMS. Adjusted odds ratios (aORs) with 95% confidence intervals (CIs) were reported, and statistical significance was set at *p* < 0.05. All statistical analyses were performed using IBM SPSS Statistics (version 26).

## Results

### Questionnaire development and validation

The questionnaire underwent a rigorous validation process prior to data collection. In the first round of expert review, several suggestions were made to enhance item clarity and alignment with the study objectives. These revisions were incorporated, and in the second round, all items achieved an I-CVI of 1.0, indicating complete expert agreement on item relevance. For face validity, 30 pregnant women, independent of the main study sample, were involved to assess clarity and cultural appropriateness. Their feedback led to minor refinements in wording. Validity and reliability were evaluated using a separate sample of 200 pregnant women. Construct validity was examined using Pearson's correlation analysis, with all item-total correlations statistically significant (*p* < 0.001) and ranging from *r* = 0.458 to 0.766 across items. Reliability testing showed acceptable internal consistency, with Cronbach’s alpha values ranging from 0.681 to 0.731 for the KAA scales. Participant demographics and the results of the validity and reliability assessments from the pilot study are presented in Multimedia Appendix 3 (Tables S2–S4, respectively).

### Study size and participant characteristics

**Fig. 1:**
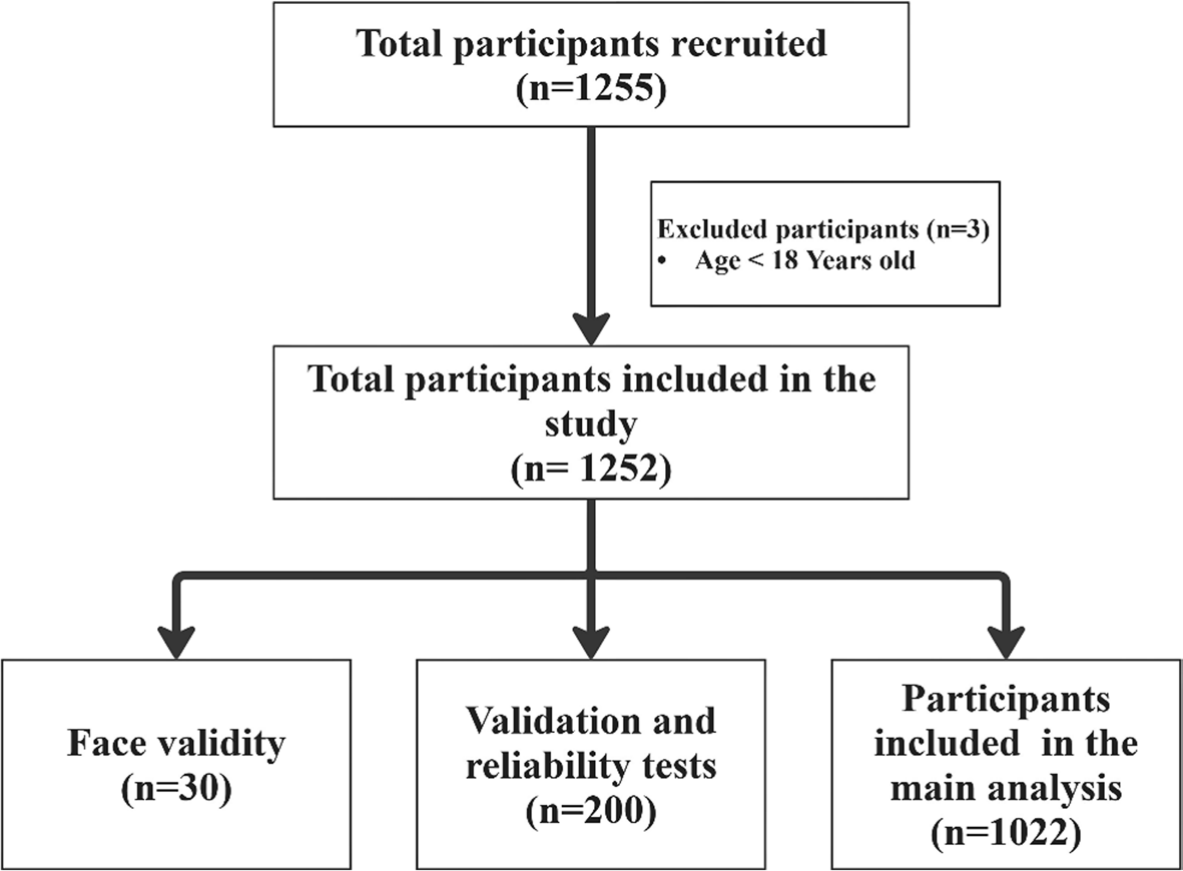
The flow diagram of the included participsant

A total of 1,255 pregnant women were initially recruited for the study. From this initial pool, three participants were excluded due to being under 18 years of age, resulting in 1,252 eligible participants. Of these eligible participants, 30 participants were allocated for face validity testing, and 200 participants for questionnaire validation and reliability testing. The remaining 1,022 pregnant women were included in the main analysis. The flowchart of participant inclusion and exclusion is presented in Fig. 1.

Among the 1,022 pregnant women included in the main analysis, most were between 25 and 34 years of age (67.8%), had attained at least a senior high school education (42%), and were unemployed (55.2%). Nearly all were married (99.3%), with slightly more residing in rural areas (51.6%) than urban (48.4%). Most were in their third trimester (61.6%) and multigravida (75.3%). Household income varied, with 31.5% earning 1–3 million Indonesian Rupiah monthly 29.1% earning 3–5 million and 22.1% earning more than 5 million. More details can be found in Table 1.

**Table 1:** Sociodemographic characteristics of participants (*n* = 1022)

Variable	Category	*n* (%)
Age (Years)	18–24	244 (23.9)
	25–34	693 (67.8)
	35–39	72 (7)
	40 and older	13 (1.3)
Education	Unschooled	85 (8.3)
	Elementary School	93 (9.1)
	Junior High School	133 (13)
	Senior High School	429 (42)
	Higher Education	282 (27.6)
Employment	Employed	458 (44.8)
	Unemployed	564 (55.2)
Marital status	Married	1015 (99.3)
	Unmarried	7 (0.7)
Monthly household income (Million Indonesian Rupiah)	Less than 1	174 (17)
	1–3	325 (31.5)
	3–5	297 (29.1)
	More than 5	226 (22.1)
Residence	Urban	495 (48.4)
	Rural	527 (51.6)
Trimester	First	175 (17.1)
	Second	217 (21.2)
	Third	630 (61.6)
Gravidity	Primigravida	252 (22.7)
	Multigravida	770 (75.3)

### Levels of knowledge, attitude, and acceptance

The KAA’s overall frequencies and their distribution across sociodemographic characteristics are summarized in Multimedia Appendix 4 (Tables S5 and S6, respectively).

### Maternal knowledge of MMS

Among the 1,022 women included in the analysis, 641 (62.7%) had good knowledge of MMS, while 381 (37.3%) demonstrated poor knowledge. A higher proportion of women with good knowledge of MMS were aged 25–34 years (45.4%), had attained at least senior high school education (31%) or higher education (19.4%), and were unemployed (34.8%). Knowledge levels were nearly evenly distributed between urban (30.8%) and rural (31.9%) residents. Good knowledge was also more prevalent among women in their second (17.5%) or third trimester (30.4%), as well as among multigravida participants (47%).

Item-level analysis revealed variable awareness and understanding of MMS. Participants generally showed strong awareness of the purpose and content of MMS, although knowledge was more limited regarding correct usage and potential side effects. Item-level responses for the knowledge domain are detailed in Table 2.

**Table 2:** Knowledge and acceptance items on MMS among Indonesian pregnant women (*n* = 1022)

No	Concepts	Items	Correct/affirmative answer (%)
Knowledge			
1	Awareness	Have you ever heard of MMS?	55.7
		What categories of nutrients are found in MMS?	62.5
2	Purpose	What is the purpose of MMS during pregnancy?	62.5
3	Correct Usage	At what stage of pregnancy is MMS recommended?	52.9
		How frequently should MMS be taken during pregnancy?	59.1
4	Side effects	What side effects do you associate with MMS?	41.7
Acceptance			
1	Willingness to consume	Would you take MMS if it were provided for free?	65.1
		Would you be willing to buy and take MMS if you had to pay for it?	56.8
		Would you be willing to replace your current pregnancy supplements (e.g., iron and folic acid tablets) with MMS?	56.3
2	Encouragement to others	Would you recommend MMS to other pregnant women?	59.3

*Abbreviations*: *MMS* multiple micronutrient supplementation

### Maternal attitude toward MMS

Most participants, 921 (90.1%), exhibited a positive attitude toward MMS, while only 101 (9.9%) had a negative attitude. Positive attitudes toward MMS were common across all demographic groups. The highest proportions were observed among women aged 25–34 years (61.8%), those with higher education (39.8%), and unemployed participants (50.9%). Rural women (45.7%) and multigravida women (67.7%). Women in the third trimester (52.1%) reported the most positive attitudes toward MMS.

When examining item-level responses, most participants agreed that nutritional supplements can help prevent complications during delivery. Most also believed that MMS offers greater nutritional benefits during pregnancy than standard IFAS. However, some hesitations emerged regarding potential barriers: fewer women felt confident about not experiencing side effects, and only a minority found it easy to remember to take MMS daily. In terms of preference, many pregnant women favored supplements, though many still leaned toward natural food sources. Detailed breakdowns of attitude responses are shown in Table 3.

**Table 3:** Attitude items on MMS among indonesian pregnant women (*n* = 1022)

No	Concepts	Items	%				
			**Strongly agree**	**Agree**	**Neutral**	**Disagree**	**Strongly disagree**
1	Beliefs	I believe nutritional supplements help prevent complications during delivery	39.4	35.1	8.7	8.5	8.2
		I believe that MMS is better than iron and folic acid supplements for improving my nutrition during pregnancy	27.5	35.4	18.1	10.9	8.1
2	Barriers	I am not worried about side effects of MMS	11.5	24.3	29.7	24.3	11.5
		I don’t find it difficult to remember to take MMS every day	10.7	23.6	23.6	30.3	11.8
3	Preference	I prefer supplements for nutrition over natural food sources	17.5	25.7	24.6	23.1	9.1

*Abbreviations*: *MMS* multiple micronutrient supplementation

### Maternal acceptance of MMS

Regarding MMS acceptance, 680 participants (66.5%) reported high acceptance, whereas 342 (33.5%) indicated low acceptance. High acceptance of MMS was most frequent among women aged 25–34 (45.6%) and those with at least senior high school (33.3%) or higher education (18.3%). Acceptance was similarly high across employed (28.3%) and unemployed (38.3%) groups, and between married and unmarried women. Women in the third trimester (33.7%) and rural residents (34.4%) reported the highest acceptance level. Multigravida women (50.2%) were also more likely to report high acceptance than primigravida participants (16.3%).

When asked about their willingness to use MMS, most participants indicated that they would take if it was provided free of charge, while a smaller proportion expressed a willingness to purchase it themselves. More than half were also open to replacing their current pregnancy supplements with MMS. Additionally, many women reported that they would recommend MMS to other pregnant women. These acceptance-related responses are presented in Table 2.

### Factors associated with knowledge, attitude, and acceptance

Bivariate associations between sociodemographic characteristics and the three outcome domains (KAA) are presented in Multimedia Appendix 4 (Table S6), while the results of the multivariable logistic regression analyses are shown in Table 4.

**Table 4:** Multivariable logistic regression analysis of study variables with knowledge, attitude and acceptance of MMS among pregnant women in Indonesia (*n* = 1022)

Variable	Category	Knowledge^$^		Attitude^¥^		Acceptance^¶^	
		**aOR (95% CI)**	***p*-value**	**aOR (95% CI)**	***p*-value**	**aOR (95% CI)**	**p-value**
Age (Years)	18–24	0.36 (0.11–1.24)	0.10	NA		NA	
	25–34	0.27 (0.08–0.89)	0.03*				
	35–39	0.60 (0.16–2.20)	0.44				
	40 and older	Reference					
Education	Elementary school	0.88 (0.46–1.71)	0.71	1.05 (0.48–2.30)	0.90	1.15 (0.61–2.16)	0.67
	Junior high school	0.46 (0.25–0.85)	0.01*	1.22 (0.59–2.51)	0.60	1.26 (0.69–2.30)	0.45
	Senior high school	0.28 (0.16–0.49)	< 0.001**	0.67 (0.32–1.14)	0.27	0.63 (0.36–1.08)	0.09
	Higher education	0.32 (0.18–0.57)	< 0.001**	0.74 (0.36–1.52)	0.41	1.01 (0.58–1.77)	0.96
	Unschooled	Reference		Reference		Reference	
Employment	Employed	NA		1.60 (1.02–2.49)	0.05*	1.17 (0.86–1.58)	0.33
	Unemployed			Reference		Reference	
Monthly household income (Million Indonesian Rupiah)	1–3	0.64 (0.42–0.98)	0.04*	0.68 (0.68–1.27)	0.23	0.71 (0.46–1.10)	0.13
	3–5	0.56 (0.34–0.87)	0.01**	0.46 (0.46–0.89)	0.02*	0.67 (0.43–1.05)	0.08
	More than 5	0.52 (0.33–0.83)	0.01**	1.08 (1.08–1.98)	0.80	1.08 (0.68–1.71)	0.75
	Less than 1	Reference		Reference		Reference	
Residence	Urban	NA		0.69 (0.44–1.08)	0.10	NA	
	Rural			Reference			
Trimester	First	0.22 (0.14–0.35)	< 0.001**	0.09 (0.02–0.39)	< 0.001**	0.58 (0.32–1.04)	0.58
	Second	0.30 (0.20–0.45)	< 0.001**	0.04 (0.01–0.30)	< 0.001**	2.13 (1.34–3.37)	< 0.001**
	Third	Reference		Reference		Reference	
Knowledge of MMS	Good	NA		0.52 (0.33–0.82)	0.01**	0.25 (0.18–0.34)	< 0.001**
	Poor			Reference		Reference	
Attitude toward MMS	Positive	0.54 (0.34–0.85)	0.01**	NA		1.00 (0.63—1.58)	0.99
	Negative	Reference				Reference	

*Abbreviations*: *MMS* multiple micronutrient supplementation, *aOR* adjusted odds ratio, *CI* Confidence interval, *NA* not applicable

^$^logistic regression analysis assessing the risk factors associated with poor maternal knowledge regarding MMS

^¥^logistic regression assessing the risk factors associated with negative attitudes toward MMS

^¶^logistic regression assessing the factors associated with low acceptance of MMS

^*^significant level 0.05

^**^significant level 0.01

In bivariate analysis, several factors were significantly associated with knowledge, including age, education, income, and trimester of pregnancy. Attitudes were significantly related to education, employment, income, residence, and trimester. Regarding MMS acceptance, significant associations were observed with education, employment, income, and trimester. Additionally, knowledge and attitude were significantly associated with each other, and both were significantly associated with acceptance. All of these variables were included in the multivariable logistic regression models to identify independent predictors.

Multivariable logistic regression analyses were conducted to identify independent predictors of poor knowledge, negative attitude, and low acceptance of MMS among pregnant women. Several factors were significantly associated with poor knowledge of MMS. Women aged 25–34 were significantly less likely to have poor knowledge than those aged 40 and older (aOR: 0.27; 95% CI: 0.08–0.89). Education level was also a significant predictor, with women who had completed junior high school (aOR: 0.46; 95% CI: 0.25–0.85), senior high school (aOR: 0.28; 95% CI: 0.16–0.49), and higher education (aOR: 0.32; 95% CI: 0.18–0.57) being less likely to have poor knowledge compared to unschooled women. Household income was inversely associated with poor knowledge, with women earning 1–3 million (aOR: 0.64; 95% CI: 0.42–0.98), 3–5 million (aOR: 0.56; 95% CI: 0.34–0.87), and more than 5 million (aOR: 0.52; 95% CI: 0.33–0.83) all being less likely to have poor knowledge than those earning less than 1 million. Women in the first (aOR: 0.22; 95% CI: 0.14–0.35) and second trimesters (aOR: 0.30; 95% CI: 0.20–0.45) were also less likely to have poor knowledge than those in the third trimester. Finally, women with a positive attitude were less likely to have poor knowledge (aOR: 0.54; 95% CI: 0.34–0.85).

Regarding attitude, household income and knowledge were significant predictors of negative attitudes toward MMS. Women with a monthly income of 3–5 million IDR were significantly less likely to report a negative attitude than those earning less than 1 million (aOR: 0.46; 95% CI: 0.46–0.89). Women in the first (aOR: 0.09; 95% CI: 0.02–0.39) and second trimesters (aOR: 0.04; 95% CI: 0.01–0.30) were also significantly less likely to have a negative attitude than those in the third trimester. Additionally, having good knowledge of MMS significantly reduced the odds of a negative attitude (aOR: 0.52; 95% CI: 0.33–0.82). Employment was also associated with attitude, as employed women were significantly more likely to report a negative attitude compared to unemployed women (aOR: 1.60; 95% CI: 1.02–2.49).

Some factors emerged as significant in the model predicting low acceptance of MMS. Low acceptance was more likely among women in the second trimester (aOR: 2.13; 95% CI: 1.34–3.37). Conversely, good knowledge of MMS was a strong protective factor, with women in the good knowledge group being significantly less likely to report low acceptance (aOR: 0.25; 95% CI: 0.18–0.34).

## Discussion

Despite longstanding recommendations for IFAS during pregnancy, national coverage in Indonesia remains low, with only 26% of pregnant women reportedly consuming the recommended ≥ 90 tablets. This suboptimal adherence has prompted a national transition toward MMS, which offers broader maternal and fetal health benefits. In this context, our study assessed the KAA of MMS among pregnant women and identified key influencing factors. While most participants demonstrated a positive attitude toward MMS, notable gaps in knowledge and acceptance persisted. Multivariable analysis revealed that age, education, income, and trimester of pregnancy were independently associated with knowledge levels. Knowledge, in turn, significantly predicted both attitude and acceptance. While sociodemographic factors such as income and trimester also influenced attitude, attitude was not significantly associated with MMS acceptance in Indonesia.

Our findings revealed that although awareness of MMS existed among many participants, substantial knowledge gaps remained, particularly concerning appropriate usage and side effects. These deficiencies were more pronounced among pregnant women with lower education levels and lower income, as well as those in later stages of pregnancy. Women in earlier trimesters were more likely to demonstrate better knowledge, reflecting increased exposure to recent antenatal counseling, as MMS is a relatively new intervention within Indonesia's maternal health system. This suggests that earlier antenatal contact plays a critical role in exposure to updated information [58, 59]. The observed association between education and knowledge is consistent with previous studies conducted in LMICs, including in Indonesia, where limited health literacy has been linked to poorer maternal nutrition knowledge [60]. Another study reported that higher educational attainment was associated with better awareness of antenatal supplements [61]. This emphasizes the need for tailored communication strategies that target lower-educated women and reinforce knowledge early in the antenatal period [62]. Moreover, attitude toward MMS also emerged as a predictor of knowledge in this study, reinforcing the notion that belief in the value of supplementation may encourage information-seeking behavior or improve retention of health information [63]. This relationship is consistent with constructs from the Health Belief Model, which posits that positive attitudes or perceived benefits can drive individuals to engage more actively in health-related learning and behaviors [64].

Despite the presence of knowledge gaps, the majority of participants in this study expressed a favorable attitude toward MMS. This finding has also been reported in previous research where favorable perceptions of antenatal supplementation were common even in the presence of limited knowledge [65]. This disconnect may reflect a general trust in health interventions and the authority of antenatal care providers within the Indonesian health system. In this study, positive attitudes were significantly more common among pregnant women with higher incomes, those who were unemployed, and those in earlier stages of pregnancy. Higher income may reflect greater financial security, which can reduce concerns about the cost or accessibility of supplementation, thereby fostering more open and favorable perceptions [66, 67]. Unemployed pregnant women may have more time and flexibility to attend antenatal visits, engage in counseling sessions, and absorb health information, all of which could contribute to a more supportive outlook toward MMS [68, 69]. Meanwhile, women in earlier trimesters may be more receptive to new information and motivated to adopt preventive behaviors at the start of pregnancy [70, 71]. This timing may also align with their first or second exposure to structured antenatal care, during which MMS counseling is more likely to occur.

In this study, the overall acceptance of MMS among pregnant women was generally high. However, multivariable analysis revealed specific factors associated with low acceptance. Women in the second trimester were more likely to report low acceptance. This finding contradicts previous assumptions that early antenatal engagement promotes higher uptake and may indicate missed opportunities for effective MMS counseling during mid-pregnancy [72–74]. One possible explanation is that women in the second trimester may face increased physical discomforts or shifting priorities that reduce focus on supplement adherence [75, 76]. Additionally, if counseling is not repeated or reinforced during this period, initial gains in awareness may diminish over time. In line with our findings, previous studies have also identified knowledge as a key predictor of acceptance [77]. Pregnant women with a better understanding of MMS, its benefits, usage, and purpose, were significantly more likely to report high acceptance. This reinforces the critical role of informed awareness in translating antenatal recommendations into health behaviors and highlights the need for targeted education and early engagement [78, 79].

This study has several strengths. It is the first to assess maternal KAA of MMS in the context of Indonesia’s national rollout of the intervention, providing timely insights during this critical transition. Additionally, the study included a substantial sample size from varied geographic and sociodemographic backgrounds, which supports exploratory insights into patterns across different subgroups. Using validated and pre-tested questionnaire further strengthens the reliability of the KAA measurements. However, the study also has several limitations. Its cross-sectional design precludes any conclusions about causality between the identified predictors and the outcomes. Moreover, the reliance on convenience sampling may have introduced selection bias and limited the representativeness of the findings. Additionally, our recruitment approach via pregnancy-related social media groups and health professional networks might have disproportionately attracted participants with superior health knowledge and higher receptivity to health interventions, potentially introducing information bias and further limiting the generalizability of the findings. Furthermore, using an online survey platform may limit participation among women with limited access to technology or lower digital literacy. Future research should consider using qualitative or mixed-methods approaches to explore deeper dimensions of MMS acceptance, particularly those related to accessibility and availability at the primary healthcare level. Such studies should also examine how supply-side barriers, socioeconomic conditions, and gender-related factors influence KAA to MMS in real-world settings. Finally, all data were self-reported, which may introduce recall or social desirability bias.

The findings of this study offer several important implications for maternal health programs in Indonesia, particularly in the context of scaling up MMS within antenatal care services. The strong association between knowledge and both attitude and acceptance underscores the need for enhanced health education strategies during pregnancy. Early and clear communication about MMS benefits, usage, and safety should be integrated into the first antenatal care visits to maximize reach and impact [80]. Given the proven influence of family members and religious leaders in shaping health behaviors, such as during vaccine introduction campaigns, involving them in MMS awareness efforts may help build community trust and reinforce positive norms [81]. The inverse relationship between employment and attitude highlights the need for more flexible and accessible service delivery models, such as workplace counseling, extended clinic hours, or digital reminders, to accommodate the needs of working women [82–84]. Additionally, the role of socioeconomic status in shaping knowledge calls for tailored messaging and outreach to lower-income populations, ensuring equitable access to information and services [85]. Moreover, strengthening the capacity of frontline healthcare providers, particularly midwives and primary care staff, to deliver consistent and culturally appropriate MMS counseling will be essential [86]. In addition, community health workers (CHWs) can play a crucial role in enhancing health promotion and outreach for MMS, particularly in underserved or rural communities [87]. Their close ties to the community and role in primary health care delivery make them well-positioned to improve awareness, dispel myths, and support adherence among pregnant women. All these actions could improve the uptake of MMS, reduce micronutrient deficiencies, and contribute to better maternal and child health outcomes.

## Conclusion

This study provides comprehensive insights into the levels and determinants of KAA of MMS among pregnant women in Indonesia. While overall attitudes toward MMS were highly positive, substantial gaps in knowledge and less-than-optimal acceptance highlight the need for strengthened health education and counseling. Key predictors of these outcomes included gestational trimester, income, employment status, and knowledge, underscoring the importance of both socioeconomic and behavioral factors in shaping maternal health behaviors. Knowledge emerged as a consistent and robust determinant across all domains, reinforcing its central role in driving uptake. Efforts to scale up MMS should prioritize reaching women in the early stages of pregnancy, when receptiveness to new health interventions is highest, and ensuring that accurate, comprehensible information is delivered through trusted channels. By addressing both informational and structural barriers, MMS programs can be more effectively integrated into maternal health services and contribute to improved nutritional and pregnancy outcomes in Indonesia and similar settings. Strategic communication, early antenatal counseling, and equitable access to MMS are crucial for successful national implementation.

## Supplementary Information

Supplementary Material 1.

Supplementary Material 2.

Supplementary Material 3.

Supplementary Material 4.

## Data Availability

The full datasets produced and analyzed during this study are not publicly available in order to safeguard participant anonymity. However, they are available from the corresponding author upon reasonable request.

## Abbreviations

aOR: Adjusted odds ratios
CI: Confidence intervals
CHWs: Community health workers
CROSS: Checklist for Reporting of Survey Studies
HIMPISI: Indonesian Society of Maternal–Fetal Medicine
I-CVI: Individual content validity index
IFAS: Iron-Folic Acid Supplements
KAA: Knowledge, attitudes, and acceptance
LMICs: Low- and middle-income countries
MMS: Multiple Micronutrient Supplementation
POGI: Indonesian Society of Obstetricians and Gynecologists
UN: United Nations
UNIMMAP: United Nations International Multiple Micronutrient Antenatal Preparation
