# Supplementary material for: Maternal perspectives on Multiple Micronutrient Supplementation (MMS) in Indonesia: a cross-sectional study of knowledge, attitudes, and acceptance
Source: BMC Public Health. 2025 Nov 19;25:4062. doi: 10.1186/s12889-025-24885-5 (PMC12629038; doi:10.1186/s12889-025-24885-5)
Supplement: Supplementary file 2 — Supplementary Material 2. [file 12889_2025_24885_MOESM2_ESM.docx]

# Multimedia Appendix 2

# Study Questionnaire (English Version)

## Section 1: Demographic Information

1. 1. Age (in years): ______
2. 2. What is the highest level of education you have completed?
    - No formal education
    - Primary school
    - Junior high school
    - Senior high school
    - Higher education
3. 3. What is your current employment status?
    - Employed
    - Unemployed
4. 4. What is your current marital status?
    - Single
    - Married
    - Divorced
5. 5. What is your average monthly household income?
    - Less than IDR 1,000,000
    - IDR 1,000,000 – 3,000,000
    - IDR 3,000,000 – 5,000,000
    - More than IDR 5,000,000
6. 6. Where do you currently live?
    - Urban area
    - Rural area
7. 7. Gestational age (in weeks): ______
8. 8. Number of previous pregnancies (excluding the current pregnancy): ______

## Section 2: Knowledge of MMS

1. 1. Have you ever heard of Multiple Micronutrient Supplementation (MMS)?
    - Yes
    - No
    - Not sure
2. 2. What categories of nutrients are found in MMS?
    - Vitamins and minerals
    - Proteins
    - Don’t know/Not sure
3. 3. What is the purpose of MMS during pregnancy?
    - To increase appetite
    - To prevent pregnancy complications and support fetal development
    - Don’t know/Not sure
4. 4. At what stage of pregnancy is MMS recommended?
    - First trimester
    - Throughout pregnancy
    - Don’t know/Not sure
5. 5. How frequently should MMS be taken during pregnancy?
    - Daily
    - Weekly
    - Don’t know/Not sure
6. 6. What side effects do you associate with MMS?
    - Diarrhea and joint pain
    - Nausea and constipation
    - Don’t know/Not sure

## Section 3: Attitudes Toward MMS

Please indicate your level of agreement with the following statements.
Response options:
1 = Strongly disagree
2 = Disagree
3 = Neutral
4 = Agree
5 = Strongly agree

1. 1. I believe nutritional supplements help prevent complications during delivery.
2. 2. I believe that MMS is better than iron and folic acid supplements for improving my nutrition during pregnancy.
3. 3. I am not worried about side effects of MMS
4. 4. I don’t find it difficult to remember to take MMS every day.
5. 5. I prefer supplements for nutrition over natural food sources.

## Section 4: Acceptance of MMS

1. 1. Would you take MMS if it were provided for free?
    - Yes
    - No
    - Not sure
2. 2. Would you be willing to buy and take MMS if you had to pay for it?
    - Yes
    - No
    - Not sure
3. 3. Would you be willing to replace your current pregnancy supplements (e.g., iron and folic acid tablets) with MMS?
    - Yes
    - No
    - Not sure
4. 4. Would you recommend MMS to other pregnant women?
    - Yes
    - No
    - Not sure
