# Supplementary material for: Maternal perspectives on Multiple Micronutrient Supplementation (MMS) in Indonesia: a cross-sectional study of knowledge, attitudes, and acceptance
Source: BMC Public Health. 2025 Nov 19;25:4062. doi: 10.1186/s12889-025-24885-5 (PMC12629038; doi:10.1186/s12889-025-24885-5)
Supplement: Supplementary file 3 — Supplementary Material 3. [file 12889_2025_24885_MOESM3_ESM.docx]

# Multimedia Appendix 3

# Table S2: Characteristics of the study participants in the validity and reliability test (n= 200)

| **Variable** | **Category** | **n (%)** |
| --- | --- | --- |
| **Age (Years)** | 18–24 | 50 (25) |
|  | 25–34 | 129 (64.5) |
|  | 35–39 | 20 (10) |
|  | 40 and older | 1(0.5) |
| **Education level** | Unschooled | 15 (7.5) |
|  | Elementary School | 20 (10) |
|  | Junior High School | 30 (15) |
|  | Senior High School | 76 (38) |
|  | Higher Education | 59 (29.5) |
| **Employment status** | Employed | 79 (39.5) |
|  | Unemployed | 121 (60.5) |
| **Marital status** | Married | 200 (100) |
|  | Unmarried | 0 |
| **Monthly household income**  **(Million Indonesian Rupiah)** | Less than 1 | 43 (21.5) |
|  | 1–3 | 54 (27) |
|  | 3–5 | 48 (24) |
|  | More than 5 | 55 (27.5) |
| **Residence** | Urban | 107 (53.5) |
|  | Rural | 93 (46.5) |
| **Trimester** | First | 30 (15) |
|  | Second | 37 (18.5) |
|  | Third | 133 (66.5) |
| **Gravidity** | Primigravida | 66 (33) |
|  | Multigravida | 134 (67) |

# Table S3: Item correlation with Pearson test (n=200)

| **Domain** | **Items** | **Coefficient validity (r)** | **p-value** |
| --- | --- | --- | --- |
| Knowledge | K1 | 0.556 | 0.000 |
|  | K2 | 0.766 | 0.000 |
|  | K3 | 0.734 | 0.000 |
|  | K4 | 0.649 | 0.000 |
|  | K5 | 0.723 | 0.000 |
|  | K6 | 0.493 | 0.000 |
| Attitude | A1 | 0.563 | 0.000 |
|  | A2 | 0.458 | 0.000 |
|  | A3 | 0.576 | 0.000 |
|  | A4 | 0.491 | 0.000 |
|  | A5 | 0.528 | 0.000 |
| Acceptance | Ac1 | 0.711 | 0.000 |
|  | Ac2 | 0.710 | 0.000 |
|  | Ac3 | 0.680 | 0.000 |
|  | Ac4 | 0.759 | 0.000 |

# Table S4: Reliability test of the items (n=200)

| **Domain** | **Cronbach’s Alpha** |
| --- | --- |
| Knowledge | 0.731 |
| Attitude | 0.692 |
| Acceptance | 0.681 |

**Note:** *Reliable if the Cronbach’s alpha is more than 0.60
