# Supplementary material for: Maternal perspectives on Multiple Micronutrient Supplementation (MMS) in Indonesia: a cross-sectional study of knowledge, attitudes, and acceptance
Source: BMC Public Health. 2025 Nov 19;25:4062. doi: 10.1186/s12889-025-24885-5 (PMC12629038; doi:10.1186/s12889-025-24885-5)
Supplement: Supplementary file 4 — Supplementary Material 4. [file 12889_2025_24885_MOESM4_ESM.docx]

# Multimedia Appendix 4

# Table S5: Frequency distribution of respondents according to the level of knowledge, attitude and acceptance (n=1022)

| **Level of KAA** | **n (%)** | | |
| --- | --- | --- | --- |
|  | **Knowledge** | **Attitude** | **Acceptance** |
| Poor/negative/low | 381 (37.3) | 101 (9.9) | 342 (33.5) |
| Good/positive/high | 641 (62.7) | 921 (90.1) | 680 (66.5) |

**Abbreviations**: KAA; knowledge, attitude and acceptance

# Table S6: Bivariate associations of sociodemographic characteristics with knowledge, attitude, and acceptance of MMS among pregnant women in Indonesia (n=1022)

| **Variable** | **Category** | **Level of Knowledge n (%)** | | | **Level of attitude n (%)** | | | **Level of acceptance n (%)** | | |
| --- | --- | --- | --- | --- | --- | --- | --- | --- | --- | --- |
|  |  | **Poor** | **Good** | **p-value** | **Negative** | **Positive** | **p-value** | **Low** | **High** | **p-value** |
| **Age**  **(Years)** | 18–24 | 109 (10.7) | 135 (13.2) | < 0.001** | 32 (3.1) | 212 (20.7) | 0.717 | 84 (8.2) | 60 (15.7) | 0.44 |
|  | 25–34 | 229 (22.4) | 464 (45.4) |  | 61 (6) | 632 (61.8) |  | 227 (22.2) | 466 (45.6) |  |
|  | 35–39 | 36 (3.5) | 36 (3.5) |  | 8 (0.8) | 64 (6.3) |  | 24 (2.3) | 48 (4.7) |  |
|  | 40 and older | 7 (0.7) | 6 (0.6) |  | 0 (0) | 13 (1.3) |  | 7 (0.7) | 6 (0.6) |  |
| **Education** | Unschooled | 62 (6.1) | 23 (2.3) | < 0.001** | 18 (1.8) | 67 (6.6) | < 0.001** | 48 (4.7) | 37 (3.6) | < 0.001** |
|  | Elementary school | 60 (5.9) | 33 (3.2) |  | 16 (1.6) | 77 (9.1) |  | 48 (4.7) | 45 (4.4) |  |
|  | Junior high school | 63 (6.2) | 70 (6.8) |  | 22 (2.2) | 111 (7.5) |  | 62 (6.1) | 71 (6.9) |  |
|  | Senior high school | 112 (11) | 317 (31) |  | 22 (2.2) | 407 (10.9) |  | 89 (8.7) | 340 (33.3) |  |
|  | Higher education | 84 (8.2) | 198 (19.4) |  | 23 (2.3) | 259 (39.8) |  | 95 (9.3) | 187 (18.3) |  |
| **Employment** | Employed | 173 (16.9) | 285 (27.9) | 0.77 | 57 (5.6) | 401 (39.2) | 0.01** | 169 (16.5) | 289 (28.3) | 0.03* |
|  | Unemployed | 208 (20.4) | 356 (34.8) |  | 44 (4.3) | 520 (50.9) |  | 173 (16.9) | 391 (38.3) |  |
| **Marital status** | Married | 379 (37.1) | 636 (62.2) | 0.63 | 101 (66.7) | 914 (89.4) | 0.38 | 341 (33.4) | 674 (65.9) | 0.28 |
|  | Unmarried | 2 (0.5) | 5 (0.8) |  | 0 (0) | 7 (0.7) |  | 1 (0.1) | 6 (0.6) |  |
| **Monthly household income**  **(Million Indonesian Rupiah)** | Less than 1 | 97 (9.5) | 77 (7.5) | < 0.001** | 28 (2.7) | 146 (15.9) | < 0.001** | 80 (7.8) | 94 (9.2) | < 0.001** |
|  | 1–3 | 109 (10.7) | 216 (21.1) |  | 25 (2.7) | 300 (29.4) |  | 89 (8.7) | 236 (34.7) |  |
|  | 3–5 | 97 (9.5) | 200 (29.1) |  | 18 (1.8) | 279 (27.3) |  | 83 (8.1) | 214 (20.9) |  |
|  | More than 5 | 78 (7.6) | 148 (14.5) |  | 30 (2.9) | 196 (19.2) |  | 90 (26.3) | 136 (20) |  |
| **Residence** | Urban | 180 (17.6) | 315 (30.8) | 0.56 | 41 (4) | 454 (44.4) | 0.08* | 167 (16.3) | 328 (32.1) | 0.86 |
|  | Rural | 201 (19.7) | 326 (31.9) |  | 60 (5.9) | 467 (45.7) |  | 175 (17.1) | 352 (34.4) |  |
| **Trimester** | First | 24 (2.3) | 151 (14.8) | < 0.001** | 2 (0.2) | 173 (16.9) | < 0.001** | 31 (3) | 144 (14.1) | < 0.001** |
|  | Second | 38 (3.7) | 179 (17.5) |  | 1 (0.1) | 216 (21.1) |  | 25 (2.4) | 192 (18.8) |  |
|  | Third | 319 (31.2) | 311 (30.4) |  | 98 (9.6) | 532 (52.1) |  | 286 (28) | 344 (33.7) |  |
| **Gravidity** | Primigravida | 91 (8.9) | 161 (15.8) | 0.66 | 23 (2.3) | 229 (22.4) | 0.64 | 85 (8.3) | 167 (16.3) | 0.92 |
|  | Multigravida | 290 (28.4) | 480 (47) |  | 78 (7.6) | 692 (67.7) |  | 257 (25.1) | 513 (50.2) |  |
| **Knowledge of MMS** | Good | NA | NA | NA | 606 (59.3) | 35 (3.4) | < 0.001** | 123 (12) | 518 (50.7) | < 0.001** |
|  | Poor | NA | |  | 315 (30.8) | 66 (6.5) |  | 219 (21.4) | 162 (15.9) |  |
| **Attitude toward MMS** | Positive | 66 (6.5) | 35 (3.4) | < 0.001** | NA | NA | NA | 289 (28.3) | 632 (61.8) | < 0.001** |
|  | Negative | 315 (30.8) | 606 (59.3) |  | NA | |  | 53 (5.2) | 48 (4.7) |  |

**Notes**: Statistical Test: Chi-square test; * Significant level 0.25; ** Significant level 0.01

**Abbreviations**: MMS, multiple micronutrient supplementation; NA, not applicable
